# Squamous Cell Carcinoma of the Thumb With Bone Invasion: A Case Report

**DOI:** 10.7759/cureus.100150

**Published:** 2025-12-26

**Authors:** Satoshi Takano, Masanori Nakayama, Soichiro Nakamura, Mitsuru Yagi

**Affiliations:** 1 Orthopedic Surgery, School of Medicine, International University of Health and Welfare (IUHW) Narita Hospital, Narita, JPN

**Keywords:** amputation, distal phalanx, marjolin’s ulcer, sentinel lymph node biopsy, squamous cell carcinoma, thumb

## Abstract

This report describes the case of a 57-year-old man who developed squamous cell carcinoma (SCC) on his right thumb 10 years after a traumatic injury. The lesion, initially misdiagnosed as osteomyelitis, persisted despite antibiotic treatment. We suspected a neoplasm, such as a Marjolin's ulcer, which is an SCC that develops long after trauma, including burn scars, and we therefore performed a biopsy of the lesion. A biopsy confirmed SCC with bone invasion but no vascular involvement. The patient underwent a proximal phalanx amputation and ipsilateral axillary sentinel lymph node biopsy (SLNB), which was negative. SCC of the hand has a higher rate of recurrence and metastasis than other sites, emphasizing the need for early diagnosis and appropriate treatment. Delayed detection, as in this case, may lead to more aggressive interventions. Surgical margins of at least 1 cm are recommended for tumors larger than 2 cm in diameter. SLNB can aid in prognostication. Physicians should be aware of the potential for SCC in post-traumatic lesions to avoid misdiagnosis and ensure timely intervention to improve patient outcomes. No recurrence was observed four years after surgery.

## Introduction

Although malignant tumors of the hand are generally rare, squamous cell carcinoma (SCC) is the most common, occurring more frequently in older men [1]. SCC accounts for 58-90% of all malignant hand tumors [1]. Risk factors for SCC include chronic inflammation, history of trauma, injuries with prolonged healing, and exposure to ultraviolet light [2]. Its appearance varies and may present as small erythematous plaques, nodules, large fungiform or necrotic masses. Diagnosis is confirmed by tumor biopsy and pathological examination [2]. Surgical resection is primary treatment for SCC [3]. Around 4-5% of patients with SCC develop lymph node metastases. Sentinel lymph node biopsy (SLNB) is used to detect micrometastases in patients without clinical and radiographic lymph node metastases [4].

This report presents a rare case of post-traumatic SCC of the hand with bone invasion, for which a thumb amputation and axillary SLNB were performed. Through this case report, we hope to familiarize local orthopedic surgeons and family physicians with the presentation of SCC on the fingers to avoid missed diagnoses.

## Case presentation

A 57-year-old man’s right thumb was, according to the patient's account, accidentally crushed between two pieces of timber, and the ulnar half of the nail was missing 10 years ago. An ulcerative lesion developed at the site of the nail defect on the thumb one year ago, which he initially self-treated by applying an over-the-counter disinfectant and monitoring the condition. He visited a local orthopedic clinic because of a persistent non-healing lesion with lumpy exudate two months before his visit to our hospital. The primary orthopedic physician suspected osteomyelitis of the distal phalanx and treated it with cephalosporin antibiotics orally for two months, and no blood tests were performed during this time. However, as the lesion did not improve and its size remained largely unchanged, the patient was referred to our hospital. The patient’s medical history was unremarkable except for the right thumb trauma.

**Figure 1:**
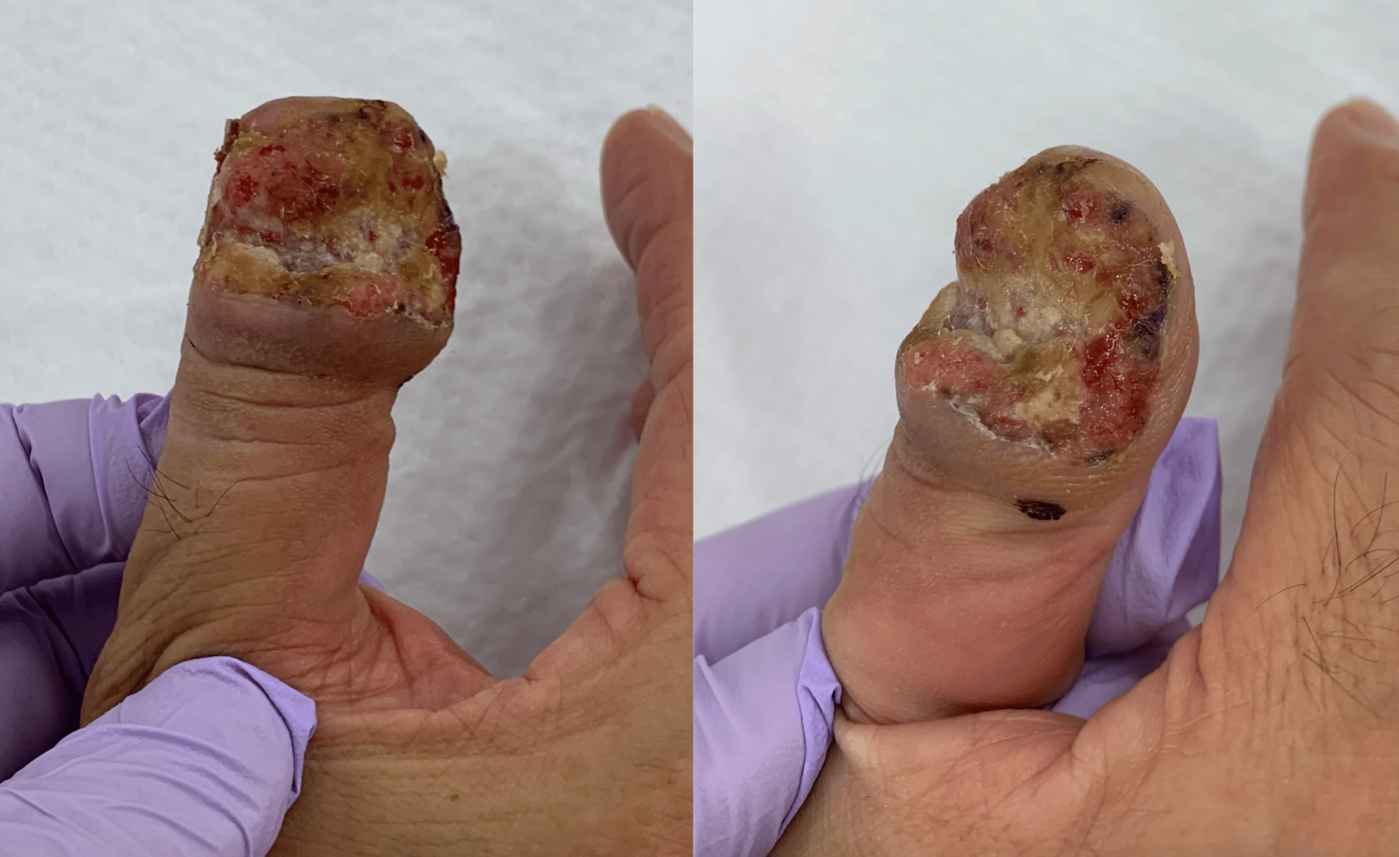
Appearance of the affected right thumb. There was a proliferative granulation at the nail defect on the ulnar side.

**Figure 2:**
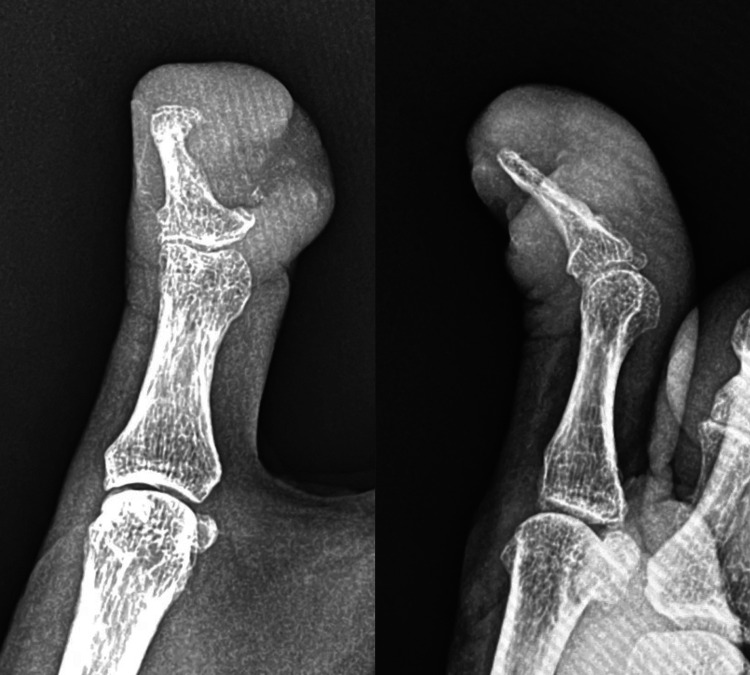
Preoperative radiographs (left: frontal view, right: lateral view).

**Figure 3:**
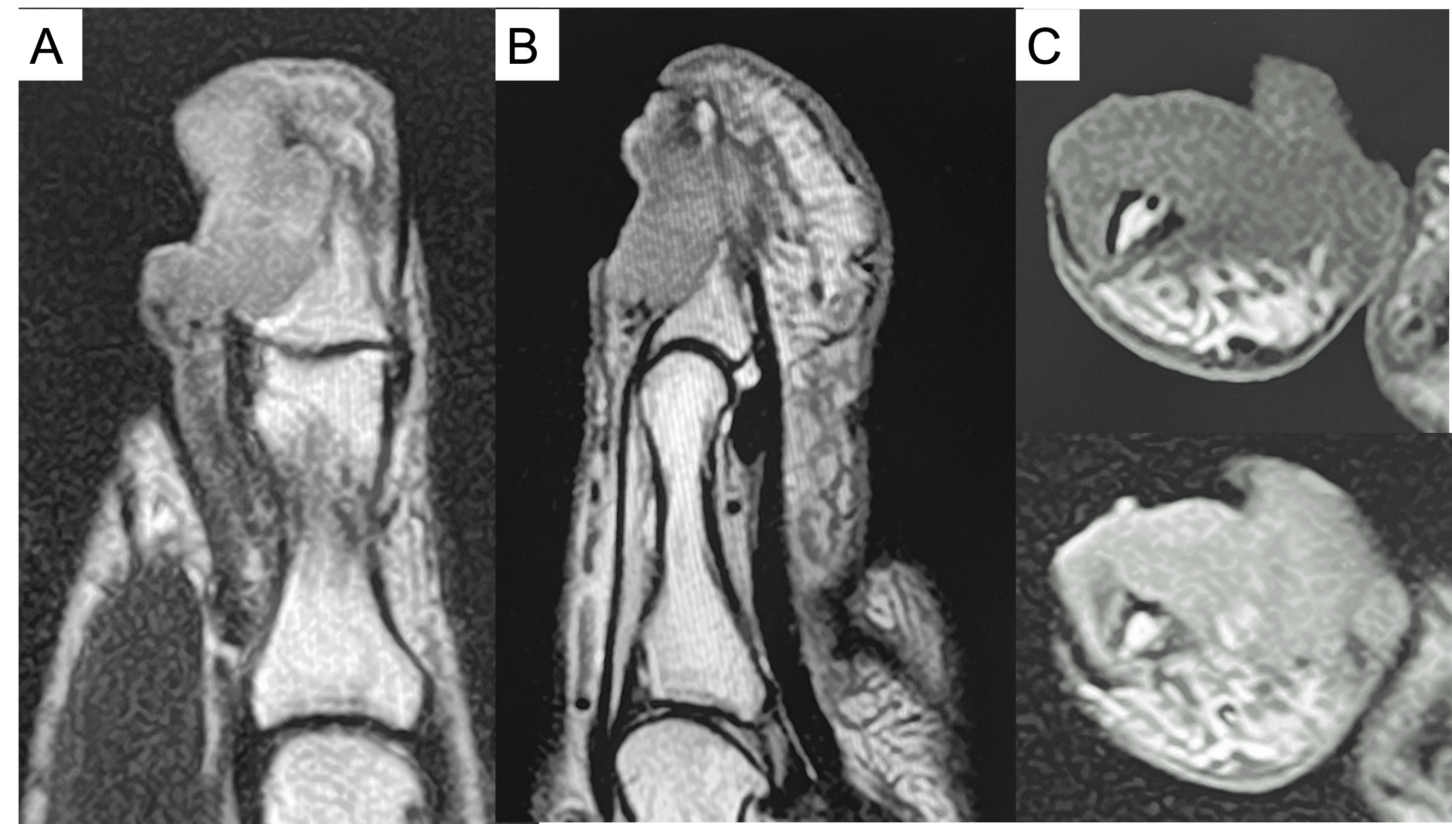
MRI images (A: coronal view of the T2-weighted image, B: sagittal view of the T2-weighted image, C: axial view (top: T1-weighted, bottom: T2-weighted).

Physical examination revealed a proliferative granulation tissue surrounding a cavity with purulent exudate at the nail defect on the ulnar side of the right thumb (Figure 1). The distal phalanx was partially exposed. There was no pain or decreased range of motion of the interphalangeal joints. His only subjective symptom was awareness of exudate from the lesion. Radiographs (Figure 2) and magnetic resonance imaging (MRI) (Figure 3) of the lesion showed a 21-mm diameter mass invading the distal phalanx. In general, malignant bone tumors typically demonstrate aggressive, ill-defined bone destruction on radiographs, whereas osteomyelitis often shows relatively localized osteolysis with reactive sclerosis and may appear normal in early stages. On MRI, malignant lesions usually exhibit heterogeneous marrow signal intensity with poorly defined margins and associated soft tissue mass formation. In contrast, osteomyelitis tends to present with more homogeneous marrow edema, diffuse enhancement, and prominent surrounding soft tissue edema or abscess formation. Cortical breakthrough with infiltrative soft tissue extension favors malignancy, while continuity with skin lesions or sinus tracts supports infection. Despite these differences, imaging findings may overlap, and histopathological confirmation is often required for definitive diagnosis [5]. Considering these factors, the imaging results suggested a malignant tumor rather than osteomyelitis.

We suspected a tumor of skin origin and referred the patient to the Department of Dermatology in our hospital. A diagnostic biopsy was performed by the dermatologist, and histopathological examination revealed SCC. Computed tomography (CT) scan of the head, chest, abdomen, and pelvis showed no evidence of distant metastasis. Surgery was jointly planned with the dermatology team.

Since a 1 cm or greater margin from tumors is required for tumors that are 2 cm or more in diameter according to the previous reports [3], the middle of the proximal phalanx bone shaft was amputated with a 1-cm margin from the tumor. The flexor pollicis longus and the extensor pollicis longus tendons were dissected. The princeps pollicis artery was cauterized, and the digital nerves were sharply dissected. The resected specimen was 38 mm in longer diameter. To facilitate future prosthetic fitting, we attempted to preserve as much of the proximal phalanx as possible, ultimately leaving 2 cm intact.

The intraoperative pathological examination showed that the resected margin was negative, and histopathology was consistent with SCC. Following our thumb resection, an ipsilateral axillary SLNB was performed by the dermatologists.

**Figure 4:**
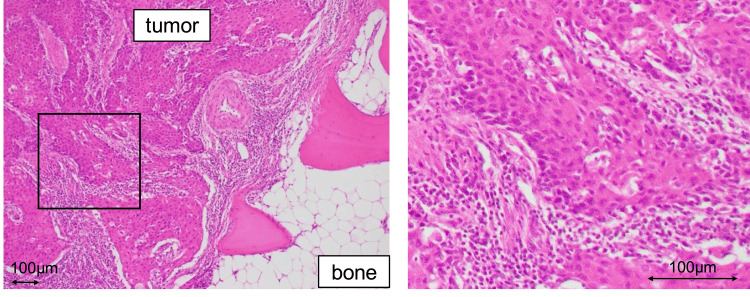
Histopathology of the resection specimen. Polygonal tumor cells with eosinophilic bodies, which show nuclear atypia, prominent nucleoli, and chromatin condensation, proliferate as nest. Intrafocal intercellular bridges and irregular keratinization were observed, and it is consistent with SCC. It is consistent with SCC and showed bone invasion. With histological staining, no obvious lymphovascular invasion was detected. SCC, squamous cell carcinoma

The final histopathological examination of the resection specimen revealed that the tumor corresponded to the macroscopically visible lesion, and it consisted of polygonal tumor cells with irregular nuclei, prominent nucleoli, increased chromatin, and eosinophilic cytoplasm that proliferated in foci and sheets (Figure 4). Consistent with the appearance of SCC, intrafocal intercellular bridges and irregular keratinization were observed. The tumor infiltrated the terminal phalanx but showed no obvious venous or lymphatic invasion. The resection margin was negative, and the SLNB result was negative.

**Figure 5:**
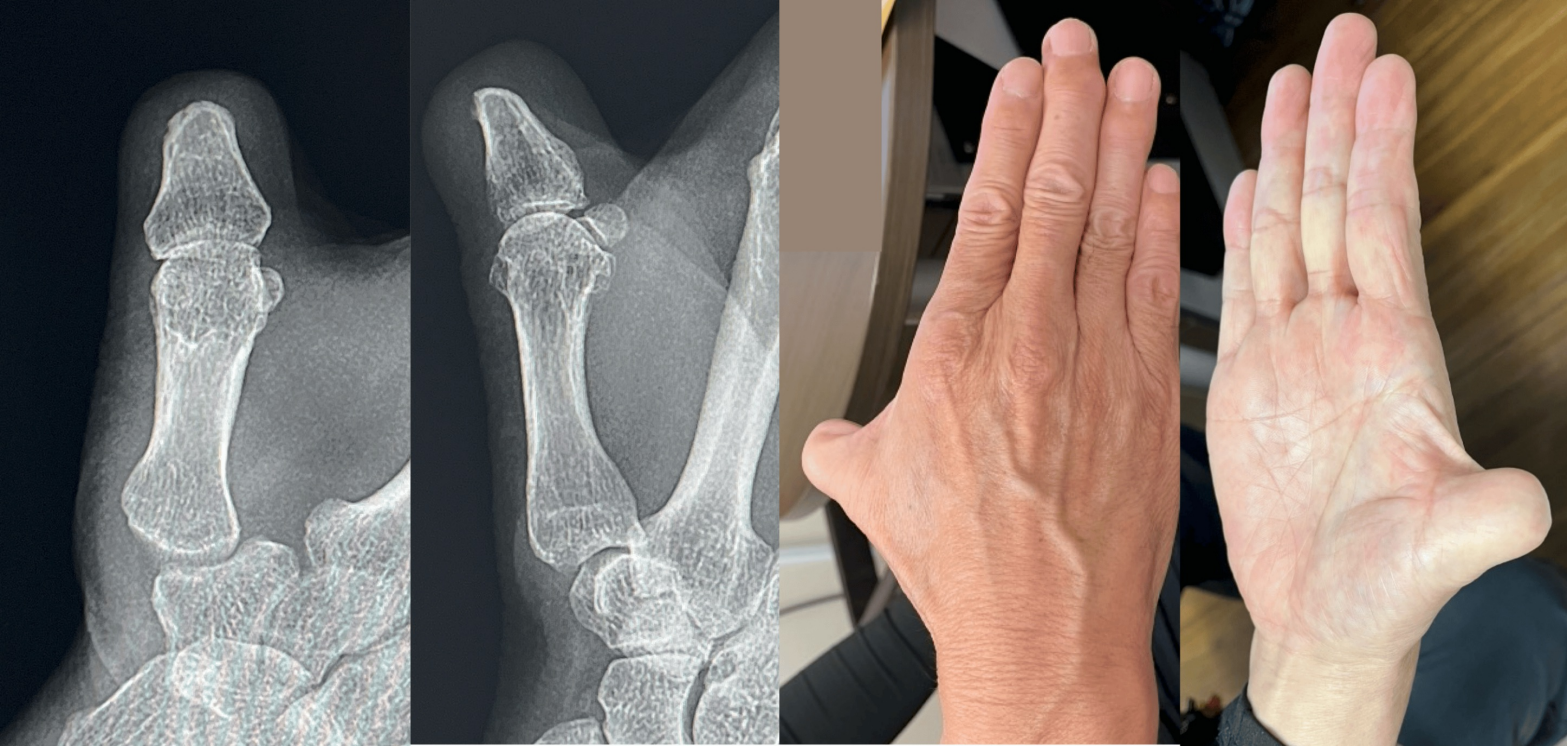
Postoperative radiographs and appearance at four years after the surgery.

Six months after the surgery, the mobility of the metacarpophalangeal joint of the right thumb was good, and the residual length of the basal phalanx was sufficient; therefore, we proposed making a prosthesis for his thumb, but the patient declined. Four years after surgery, no apparent recurrence or distant metastasis was observed on annual physical examinations, blood tests, and radiological examinations (Figure 5).

## Discussion

Cutaneous SCC arising in chronically injured or scarred tissue has been well documented in the literature. Among these, Marjolin’s ulcers represent a classical entity of SCC developing in long-standing traumas, including burn scars and chronic ulcers, often decades after the initial injury. Several reports have described such cases occurring in the extremities, particularly the hands, with latent periods ranging from 5 to over 50 years following trauma [6,7,8]. Karakozis et al. reported a case of SCC arising at an amputation stump 52 years after traumatic limb loss [9], and additional reports have documented SCC originating within long-standing traumatic or surgical scars, including keloid tissue and amputation sites [10,11]. These findings suggest that chronic inflammation, repetitive irritation, and continuous tissue remodeling in areas of previous injury may induce malignant transformation, regardless of whether the original insult was thermal or non-thermal. Our case is considered to be equivalent to such cases, representing SCC that developed after a long latent period at the site of previous non-burn trauma.

A neoplastic lesion on the hand that is strongly suspected to be malignant with bone invasion should be considered as a possible SCC [1]. This patient was diagnosed with osteomyelitis of the distal phalanx of the thumb by the primary orthopedic physician and was treated with wound care and antibiotics. It is sometimes difficult to visually distinguish between osteomyelitis and SCC, and, thus, a careful examination of characteristic imaging findings is crucial. If the diagnosis is uncertain, especially in patients with a history of trauma, a biopsy should be performed to make a proper diagnosis. The diagnosis could have been made earlier if the primary care physician had an increased index of suspicion for SCC. It is undisputed that early diagnosis and treatment of malignant tumors are essential, and it is crucial to educate local orthopedists and family physicians about this disease. Although there are reports of other diseases on the thumb that are difficult to differentiate from SCC, such as infectious granuloma and superficial acral fibromyxoma [12,13], these conditions have been initially misdiagnosed as SCC. Since these are all benign lesions, a strict differential diagnosis must be made with biopsy, if necessary.

The five-year recurrence and metastasis rates for SCC on the hand are higher than that for SCC at other sites [14]. Askari et al. reported that SCC on the hand had a mean recurrence rate of 38%, a metastasis rate of 4%, and an overall survival rate of 88% at 4.1 years [1]. Lecerf et al. specifically noted a 30.6% recurrence rate for nail unit SCC on the hand, with no overt metastases [15]. Irmak et al. reported that the recurrence and metastasis rates were increased (16.7% vs. 9.7% and 33% vs. 6.5%, respectively) for hand SCCs that were 2 cm or more in diameter compared to lesions that were less than 2 cm at 5 years of follow-up [16]. Therefore, early diagnosis and treatment of SCC on the hand, while its diameter is still small, are crucial. Surgical margins for SCC on the hand should be 5 mm or more for tumors less than 2 cm in diameter. A margin of 1 cm or greater is required for tumors that are 2 cm or more in diameter or those that are ulcerative [3]. Indications for amputation of the thumb or fingers include extensive soft tissue involvement, bone invasion, patient preference, and contraindications to reconstructive surgery [14]. In general, radiotherapy is not used for curative local control of SCC on the hands because of the high rate of radiation necrosis [14].

Although the effect of SLNB on the management and outcome of patients with cutaneous SCC remains unclear, SLNB may effectively detect potential lymph node metastasis and may aid in prognostication [4]. Takahashi et al. reported on 26 patients with SCC on the hand, with a three-year survival rate of 100% for SLNB-negative patients and only 20.8% for SLNB-positive patients [17]. Schmitt et al. concluded that SLNB can be considered for SCCs greater than 2 cm in diameter [4]. European guidelines do not currently recommend SLNB as standard practice [18], but they note that it may be considered for high-risk cases. Furthermore, a meta-analysis of head and neck cancers indicates that SLNB is effective in high-risk cases [19]. This case was considered high risk because the tumor diameter was greater than 2 cm. After consulting with a dermatologist and determining that the patient was young and needed an evaluation for metastasis, SLNB was performed with the patient's consent.

## Conclusions

Since SCCs on the hand have a more severe prognosis than those on other regions, early diagnosis and appropriate treatment are necessary to ensure better outcomes. Close follow-up is also required after primary treatment to avoid recurrence and distant metastasis.
